# Exploring Patterns and Associated Factors of Physical Inactivity in Adolescents: A Cross‑Sectional Study

**DOI:** 10.1002/hsr2.72926

**Published:** 2026-08-19

**Authors:** Claire Chenwen Zhong, Zehuan Yang, Mingtao Chen, Zhaojun Li, Vera M. W. Keung, Amelia Lo, Calvin Cheung, Junjie Huang, Martin C. S. Wong

**Affiliations:** ^1^ The Jockey Club School of Public Health and Primary Care, Faculty of Medicine The Chinese University of Hong Kong Hong Kong SAR China; ^2^ Centre for Health Education and Health Promotion, Faculty of Medicine The Chinese University of Hong Kong Hong Kong SAR China

**Keywords:** adolescence, health behaviors, mental toughness, physical inactivity

## Abstract

**Background and Aims:**

Physical inactivity is highly prevalent among adolescents and poses long‐term health risks. Understanding how individual characteristics are associated with inactivity may help inform the development of tailored interventions. This study examined the associations between adolescents' individual‐level factors and physical inactivity in Hong Kong.

**Methods:**

A cross‐sectional survey was conducted among Secondary 1–4 students in Hong Kong. A self‐administered questionnaire assessed physical inactivity and potential factors, including sociodemographic characteristics, health behaviors, health literacy, and mental toughness. Univariable and multivariable logistic regression analyses were conducted to identify potential factors associated with physical inactivity.

**Results:**

Among the 1423 adolescents, only 12.9% (*n* = 184) achieved the recommended level of physical activity (≥ 60 min of moderate‐to‐vigorous physical activity per day), while 87.1% (*n* = 1239) did not. Multivariable logistic regression showed that being over 14 years old and spending excessive screen time on electronic games were positively associated with physical inactivity. Conversely, male sex, sufficient vegetable intake, excessive screen time on social media, and higher mental toughness were inversely associated with physical inactivity. Smoking and alcohol use were also inversely associated with physical inactivity, and were reported by 1.6% and 8.4% of participants, respectively.

**Conclusion:**

A high level of physical inactivity was observed among adolescents in this study. The observed associations with age, sex, electronic gaming, vegetable intake, and mental toughness may inform the design and evaluation of school‐ and community‐level physical activity initiatives.

**Abbreviations**
aORs: adjusted odds ratios
BMI: body mass index
CHEHP: Centre for Health Education and Health Promotion
CIs: confidence intervals
FAS II: Family Affluence Scale II
GOF: goodness of fit
GSHS: Global School‐Based Student Health Survey
HELMA: Health Literacy Measure for Adolescents
MTS‐A: Mental Toughness Scale for Adolescents
MVPA: moderate‐to‐vigorous physical activity
ORs: odds ratios
SES: socioeconomic status
VIF: variance inflation factor
WHO: World Health Organization

## 1 Introduction

Physical activity refers to any body movement produced by skeletal muscles that requires the expenditure of energy [1]. According to the recommendations from the World Health Organization (WHO), children and adolescents aged 5–17 years are recommended to engage in at least 60 min of moderate‐to‐vigorous‐intensity physical activity per day, while adults should engage in at least 150–300 min of moderate‐intensity physical activity or 75–150 min of vigorous‐intensity physical activity or an equivalent combination per week [1]. Despite these guidelines, research indicates a concerning trend of physical inactivity among populations worldwide. Globally, 31.3% of adults did not meet the recommended levels of physical activity [2], and a study involving 1.6 million adolescents showed that 81.0% were classified as physically inactive [3]. These findings highlighted the potential public health crisis posed by physical inactivity, especially in the adolescent population.

Evidence gaps regarding physical inactivity among adolescents remain. While there is limited evidence linking physical inactivity to short‐term health outcomes in this age group [4], substantial research has documented the long‐term effects on both physical health and mental wellbeing. For example, a longitudinal study involving 17,415 subjects in the United Kingdom noted that individuals who were physically inactive during adolescence had a higher risk of depressive symptoms in adulthood [5]. Similarly, another longitudinal study involving 1961 subjects found that individuals who were physically inactive during adolescence had a higher risk of cardiometabolic disease in adulthood than those who were physically active during that period [6]. Importantly, evidence suggested that healthy behaviors such as patterns of physical activity during adolescence play an important role in the development of an individual's lifelong healthy behaviors, as patterns of behavior at this stage may persist into adulthood and have long‐term effects on future health [7]. As such, physical inactivity during adolescence may lead individuals into a long‐term cycle of poor health. This emphasizes the need for early intervention and focused attention on physical inactivity among adolescents to prevent health issues in adulthood.

Despite efforts to promote physical activity among adolescents, the effectiveness of many interventions has been limited, whereas targeted interventions for specific groups appear to be more effective [8]. This highlights the significance of individual‐level characteristics among adolescents in determining the effectiveness of such interventions. However, studies on predictors of physical inactivity in adolescents have focused mainly on Western cultural contexts, leaving a gap in understanding how cultural differences may affect these predictors in regions like Asia [9, 10, 11]. Population‐level surveillance further showed that Hong Kong consistently performs among the lowest globally in overall physical activity, with only about 25% of young people meeting moderate‐to‐vigorous physical activity (MVPA) guidelines [12]. Additionally, cross‑country analyses revealed substantial variation in adolescent physical activity across 52 countries, highlighting the importance of context‐specific evidence [13]. Limited local research on individual‐level determinants further reinforces the importance of studying physical inactivity within Hong Kong's specific sociocultural context [14]. Therefore, this study aimed to examine the associations of sociodemographic characteristics, health literacy, health behaviors, and mental toughness with physical inactivity among adolescents in Hong Kong, thereby strengthening the local evidence base and informing the design and evaluation of targeted interventions in the future.

## 2 Methods

The study was designed and reported in accordance with the Strengthening the Reporting of Observational Studies in Epidemiology guidelines [15]. Ethical approval was obtained from the Survey and Behavioural Research Ethics Committee of The Chinese University of Hong Kong (CUHK) (SBRE‐24‐0029).

### 2.1 Setting and Participants

This school‐based cross‐sectional study was conducted in secondary schools across Hong Kong between September and November 2024. Invitations were sent to all secondary schools to participate in the “Health Education Built‐on Project” coordinated by the Centre for Health Education and Health Promotion (CHEHP), CUHK. Schools participating in the project were then invited to complete the questionnaire survey, forming a convenience sample based on the existing CHEHP‐school network. All students enrolled in Grades 1–4 of secondary schools in Hong Kong were considered potentially eligible for participation. Under the Hong Kong education system, students at these grade levels are typically aged 12–16 years [16].

### 2.2 Data Collection

**Figure 1:**
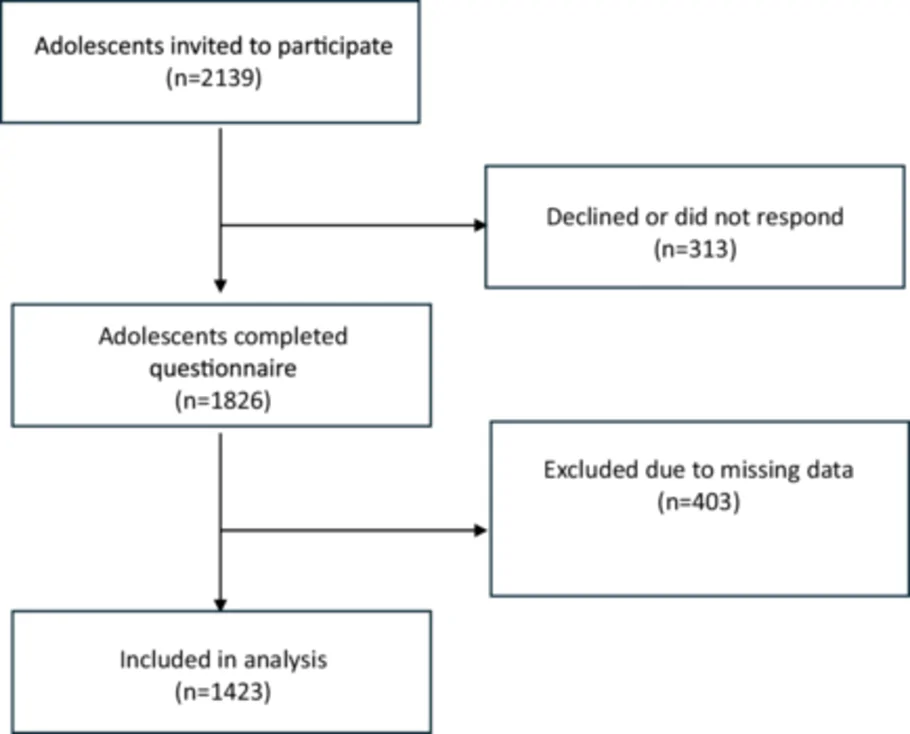
Flowchart of participant recruitment and inclusion.

A total of 440 secondary schools in Hong Kong were invited, of which 20 agreed to participate, yielding a school response rate of 4.5%. Detailed information about the study and its objectives was provided via email. The participating schools were located across 11 of the 18 geographic clusters in Hong Kong, thereby capturing adolescents from a broad range of districts. School consent was obtained through a project application form. Informed consent to participate in the study was obtained from all students, and from their parents or legal guardians for participants under 16. Two templates of parent consent forms were provided to participating schools, allowing them to select either an opt‐in or opt‐out approach for collecting parental consent based on their specific circumstances. After obtaining consent from the participating schools, paper‐based questionnaires were distributed to students during class time. Trained teachers provided standardized instructions and were available to answer students' questions throughout the process. Students were informed of the voluntary and anonymous nature of the study and their right to withdraw at any time without consequence. A total of 2139 adolescents were invited to participate in the study, of whom 1826 completed the questionnaire, yielding a response rate of 85.37%, while 313 declined or did not respond. Among those who completed the questionnaire, 403 were excluded due to missing data. Consequently, 1423 adolescents were included in the final analysis (Figure 1). No incentive was provided for participation. Completed questionnaires were sealed in envelopes and returned directly to the research team, without being accessed by teachers or school administrators. To ensure confidentiality, all personal identifiers were removed, and no identifying information was linked to the data set.

### 2.3 Survey Instruments and Measures

The primary outcome of this study was physical inactivity, defined as engaging in less than 60 min of MVPA per day, in accordance with WHO recommendations for adolescents. The measurement of physical activity was adapted from the Global School‐Based Student Health Survey (GSHS) developed by WHO [17]. Its reliability in measuring physical inactivity and sedentary behavior among school children has been validated in multiple studies across diverse countries [18, 19, 20, 21]. Physical inactivity was assessed using a single self‑reported item: “In the past 7 days, how much time did you spend engaging in moderate‑to‑vigorous physical activity?”

To explore factors associated with physical inactivity, a self‐administered questionnaire was distributed, capturing multidimensional information relevant to adolescents' health behaviors and psychosocial characteristics. The questionnaire included the following domains: (i) Demographic and Family Background: Participants reported their age, sex, and grade level. Socioeconomic status (SES) was assessed using the Family Affluence Scale II (FAS II), which evaluates material assets and recent travel frequency to derive a composite score of affluence [22]. (ii) Health Literacy: Health literacy was assessed using the validated Health Literacy Measure for Adolescents (HELMA) [23, 24]. This instrument covers a broad range of competencies, including information access, comprehension, appraisal, communication, self‐efficacy, and numeracy. Each item was rated on a 5‐point Likert scale, with higher scores indicating greater health literacy. Health literacy was dichotomized into low and high based on the median HELMA scores [24]. (iii) Health‐Related Behaviors: Students were asked to report their lifestyle patterns across dietary habits (e.g., daily fruit and vegetable consumption, frequency of breakfast intake). Participants who reported consuming fewer than 3 servings (i.e., 1.5 bowls) of vegetables and fewer than 2 servings of fruit per day over the past 7 days were classified as having insufficient vegetable and insufficient fruit intake, respectively, according to the recommendations of the Hong Kong Centre for Health Protection [25, 26]; sleep (average hours of sleep per night). Sufficient sleep was defined as obtaining at least 8 h of sleep per day, consistent with international health recommendations [27]; screen time (hours spent on video, social media, and gaming during weekdays and weekends). Excessive screen time was defined as spending more than 2 h per day on these activities, as recommended by existing guidelines [26]; substance use (tobacco and alcohol use in the past 30 days); and hygiene practices. (iv) Psychological Attributes: Mental toughness was measured using the Mental Toughness Scale for Adolescents (MTS‐A), an 18‐item instrument evaluating domains such as emotional control, interpersonal confidence, commitment, and challenge orientation [28]. For analytical purposes, mental toughness scores were dichotomized into “low” and “high” based on the sample median to differentiate participants with varying psychological profiles and to facilitate group comparisons [29]. A higher score reflected a higher level of mental toughness. The use of median cut‑points is a widely used pragmatic approach in epidemiologic and population‑based studies when the primary objective is to compare relative levels of risk across groups rather than to model precise dose–response relationships [30, 31]. While dichotomization may involve some loss of information and reduced statistical power, the median provides an objective, prespecified, and reproducible threshold that avoids data‑driven optimization [30, 31]. Sensitivity analyses using continuous measures were also conducted to assess the robustness of the findings.

### 2.4 Data Analysis

Statistical analyses and reporting were guided by published recommendations on the analysis, reporting, and interpretation of clinical research [32]. Continuous variables were summarized as means and standard deviations (SDs), and their distributions were examined using skewness, kurtosis, and visual inspection of *Q*–*Q* plots for descriptive purposes. Categorical variables were described as frequencies and percentages. Group differences were assessed using independent *t*‐tests for continuous variables and *χ* ^2^ tests for categorical variables. These *t*‐tests and *χ* ^2^ tests were conducted to describe sample characteristics and explore group differences rather than for formal hypothesis testing. Logistic regression analyses were conducted to estimate odds ratios (ORs) and 95% confidence intervals (CIs) of factors associated with physical inactivity [33]. Univariable models were used to describe crude associations. Variables were included in the multivariable model based on prior evidence and conceptual relevance, consistent with established epidemiologic principles [34, 35]. Conceptually important covariates were selected a priori based on prior literature, including sociodemographic factors (e.g., age and sex), dietary behaviors (e.g., fruit and vegetable intake), psychosocial factors, and health literacy, which have been consistently linked to adolescent physical activity [3, 24, 36, 37, 38, 39, 40, 41, 42]. The rationale for selecting these covariates is provided in Supporting Information S2: Appendix 1. Participants with missing data on variables included in the analyses were excluded; therefore, a complete‐case analysis was conducted. Multicollinearity was assessed using variance inflation factors (VIF) [43]. As a sensitivity analysis, multilevel logistic regression models were fitted with school included as a random intercept to account for clustering within schools. Results were presented as adjusted odds ratios (aORs) with 95% CIs. Hosmer–Lemeshow test was used to assess the goodness‐of‐fit of the primary multivariable logistic regression model [33]. The multilevel logistic regression model was conducted as a sensitivity analysis [33]. All statistical tests were two‑sided, with a priori statistical significance set at *p* < 0.05. The main analyses were specified a priori, and no post hoc subgroup analyses were conducted. R (version 4.2.1) was used for all statistical analyses.

## 3 Results

### 3.1 Participant Characteristics

A total of 1423 participants were included in the final analysis. Among these adolescents, only 12.9% (184/1,423) met the recommended level of physical activity (≥ 60 min of moderate‐to‐vigorous activity per day), while the majority, 87.1% (1239/1423), did not meet this recommendation. Table 1 summarizes the participants' characteristics by physical activity level. Among the 1423 participants, only 1.6% (23/1423) of students reported smoking and 8.4% (119/1423) reported alcohol use.

**Table 1:** Participant characteristics by physical activity level.

	Overall (*N* = 1423)	Sufficient physical activity = Yes (*N* = 184) *n* (row%)	Sufficient physical activity = No (*N* = 1239) *n* (row%)	*p*
Age group				0.098
< 13 years	604 (42.4%)	91 (15.0%)	513 (85.0%)	
13–14 years	405 (28.5%)	49 (12.1%)	356 (87.9%)	
> 14 years	414 (29.1%)	44 (10.6%)	370 (89.4%)	
Gender				**< 0.001**
Female	802 (56.4%)	60 (7.5%)	742 (92.5%)	
Male	621 (43.6%)	124 (20.0%)	497 (80.0%)	
SES [0–9]				0.222
Low [0–2]	236 (16.6%)	24 (10.2%)	212 (89.8%)	
Medium [3–5]	697 (49.0%)	88 (12.6%)	609 (87.4%)	
High [6–9]	490 (34.4%)	72 (14.7%)	418 (85.3%)	
Smoking				**0.027**
No	1400 (98.4%)	177 (12.6%)	1,223 (87.4%)	
Yes	23 (1.6%)	7 (30.4%)	16 (69.6%)	
Alcohol drinking				**0.021**
No	1304 (91.6%)	160 (12.3%)	1,144 (87.7%)	
Yes	119 (8.4%)	24 (20.2%)	95 (79.8%)	
Having breakfast every day				**0.035**
No	726 (51.0%)	80 (11.0%)	646 (89.0%)	
Yes	697 (49.0%)	104 (14.9%)	593 (85.1%)	
Sufficient vegetable intake				**0.007**
No	1225 (86.1%)	146 (11.9%)	1079 (88.1%)	
Yes	198 (13.9%)	38 (19.2%)	160 (80.8%)	
Sufficient fruit intake				**0.021**
No	1193 (83.8%)	143 (12.0%)	1,050 (88.0%)	
Yes	230 (16.2%)	41 (17.8%)	189 (82.2%)	
Sufficient sleep				**0.040**
No	792 (55.7%)	89 (11.2%)	703 (88.8%)	
Yes	631 (44.3%)	95 (15.1%)	536 (84.9%)	
Excessive screen time on video				> 0.999
No	595 (41.8%)	77 (12.9%)	518 (87.1%)	
Yes	828 (58.2%)	107 (12.9%)	721 (87.1%)	
Excessive screen time on electronic games				**0.037**
No	683 (48.0%)	102 (14.9%)	581 (85.1%)	
Yes	740 (52.0%)	82 (11.1%)	658 (88.9%)	
Excessive screen time on social media				0.119
No	691 (48.6%)	79 (11.4%)	612 (88.6%)	
Yes	732 (51.4%)	105 (14.3%)	627 (85.7%)	
Self‐reported obesity				0.110
No	943 (66.3%)	132 (14.0%)	811 (86.0%)	
Yes	480 (33.7%)	52 (10.8%)	428 (89.2%)	
MTS‐A				**< 0.001**
Low	784 (55.1%)	71 (9.1%)	713 (90.9%)	
High	639 (44.9%)	113 (17.7%)	526 (82.3%)	
Health literacy level				**0.014**
Low	727 (51.1%)	78 (10.7%)	649 (89.3%)	
High	696 (48.9%)	106 (15.2%)	590 (84.8%)	

*Note:* Values are presented as *n* (row %). Sufficient physical activity: moderate/vigorous exercise ≥ 60 minutes every day; Physical inactivity: moderate/vigorous exercise < 60 minutes every day; Sufficient vegetable intake: had ≥ 1.5 bowls of vegetables every day; Sufficient fruit intake: had ≥ 2 servings of fruit every day; Sufficient sleep: sleep at least 8 h; MTS‐A: Mental Toughness Scale for Adolescents, the scale [18–72] is composed of six dimensions, including challenge [3–12], commitment [3–12], emotion control [3–12], life control [3–12], confidence in abilities [3–12], and interpersonal confidence [3–12], with a higher score indicating higher mental toughness. Based on the sample median, mental toughness was dichotomized into low (≤ 46) and high (> 46); Health literacy level: scores for evaluating personal competencies for the access to, understanding of, appraisal of and application of health information in order to make sound decisions in everyday life. Based on the sample median, health literacy was categorized into low (44–144) and high (145–220). Statistically significant associations (*p* < 0.05) are shown in bold.

Abbreviation: SES, socioeconomic status.

Bivariate comparisons revealed significant differences in physical activity patterns across multiple variables. Female adolescents exhibited a notably higher proportion of physical inactivity than males (92.5% [742/802] vs. 80.0% [497/621], *p* < 0.001). A higher level of physical inactivity was also observed among those with insufficient fruit intake (88.0% [1050/1193] vs. 82.2% [189/230], *p* = 0.021), insufficient sleep (88.8% [703/792] vs. 84.9% [536/631], *p* = 0.040), and excessive time spent on electronic games (88.9% [658/740] vs. 85.1% [581/683], *p* = 0.037). The MTS scores were approximately normally distributed (mean = 46.19, SD = 8.04; median = 46, IQR = 10; skewness = 0.15, kurtosis = 3.56), ranging from 21 to 72. Similarly, the health literacy level scores were approximately normally distributed (mean = 144.74, SD = 29.99; median = 144, IQR = 39; skewness = −0.27, kurtosis = 3.47), ranging from 44 to 220 (Supporting Information S1: Table 1). Visual inspection of the *Q*–*Q* plots also suggested that both variables were approximately normally distributed (Supporting Information S1: Figure 1). Based on the sample distribution, mental toughness scores were dichotomized at the median into low (≤ 46) and high (> 46), while health literacy was similarly categorized into low (44–144) and high (145–220). Adolescents with lower mental toughness (90.9% [713/784] vs. 82.3% [526/639], *p* < 0.001) and lower health literacy (89.3% [649/727] vs. 84.8% [590/696], *p* = 0.014) also showed higher levels of physical inactivity. A higher prevalence of physical inactivity was observed among adolescents who did not report smoking (87.4% [1223/1400] vs. 69.6% [16/23], *p* = 0.027) or alcohol use (87.7% [1144/1304] vs. 79.8% [95/119], *p* = 0.021) compared with those who reported these behaviors.

### 3.2 Factors Associated With Physical Inactivity

Univariable logistic regression analysis (Table 2) identified 11 factors associated with physical inactivity. Age > 14 years (OR = 1.492, 95% CI: 1.016–2.190, *p* = 0.041) and excessive screen time on electronic games were positively associated with physical inactivity (OR = 1.409, 95% CI: 1.032–1.923, *p* = 0.031) compared to their counterparts. In contrast, being male (OR = 0.324, 95% CI: 0.233–0.450, *p* < 0.001), having healthier behaviors such as daily breakfast consumption (OR = 0.706, 95% CI: 0.517–0.965, *p* = 0.029), sufficient vegetable intake (OR = 0.570, 95% CI: 0.384–0.845, *p* = 0.005) and sufficient fruit intake (OR = 0.628, 95% CI: 0.429–0.918, *p* = 0.016), and sufficient sleep (OR = 0.714, 95% CI: 0.524–0.974, *p* = 0.034) were associated with lower odds of physical inactivity. Likewise, adolescents with higher levels of mental toughness (OR = 0.464, 95% CI: 0.337–0.637, *p* < 0.001) and health literacy (OR = 0.669, 95% CI: 0.489–0.915, *p* = 0.012) had lower odds of physical inactivity. In addition, smoking (OR = 0.331, 95% CI: 0.134–0.815, *p* = 0.016) and alcohol consumption (OR = 0.554, 95% CI: 0.343–0.892, *p* = 0.015) were inversely associated with physical inactivity.

**Table 2:** Factors associated with physical inactivity.

	OR (95% CI)	*p*	aOR (95% CI)	*p*
Age group				
< 13	Reference		Reference	
13–14	1.289 (0.888–1.871)	0.182	1.483 (0.992–2.217)	0.055
> 14	1.492 (1.016–2.190)	**0.041**	1.627 (1.068–2.478)	**0.023**
Gender				
Female	Reference		Reference	
Male	0.324 (0.233–0.450)	**< 0.001**	0.284 (0.198–0.407)	**< 0.001**
SES				
Low	Reference		Reference	
Medium	0.783 (0.486–1.263)	0.317	0.83 (0.503–1.371)	0.467
High	0.657 (0.402–1.073)	0.094	0.75 (0.444–1.267)	0.282
Smoking				
No	Reference		Reference	
Yes	0.331 (0.134–0.815)	**0.016**	0.351 (0.125–0.988)	**0.047**
Alcohol drinking				
No	Reference		Reference	
Yes	0.554 (0.343–0.892)	**0.015**	0.507 (0.293–0.877)	**0.015**
Having breakfast every day				
No	Reference		Reference	
Yes	0.706 (0.517–0.965)	**0.029**	0.86 (0.611–1.209)	0.385
Sufficient vegetable intake				
No	Reference		Reference	
Yes	0.570 (0.384–0.845)	**0.005**	0.599 (0.389–0.921)	**0.020**
Sufficient fruit intake				
No	Reference		Reference	
Yes	0.628 (0.429–0.918)	**0.016**	0.801 (0.526–1.219)	0.300
Sufficient sleep				
No	Reference		Reference	
Yes	0.714 (0.524–0.974)	**0.034**	0.824 (0.585–1.160)	0.267
Excessive screen time on video				
No	Reference		Reference	
Yes	1.002 (0.732–1.371)	0.992	0.956 (0.669–1.367)	0.806
Excessive screen time on electronic games				
No	Reference		Reference	
Yes	1.409 (1.032–1.923)	**0.031**	1.884 (1.309–2.711)	**0.001**
Excessive screen time on social media				
No	Reference		Reference	
Yes	0.771 (0.564–1.054)	0.102	0.510 (0.353–0.736)	**< 0.001**
Self‐reported obesity				
No	Reference		Reference	
Yes	1.340 (0.952–1.885)	0.093	1.181 (0.824–1.694)	0.365
MTS‐A				
Low	Reference		Reference	
High	0.464 (0.337–0.637)	**< 0.001**	0.643 (0.451–0.915)	**0.014**
Health literacy level				
Low	Reference		Reference	
High	0.669 (0.489–0.915)	**0.012**	0.764 (0.538–1.084)	0.131

*Note:* Physical inactivity: moderate/vigorous exercise < 60 minutes every day; Sufficient vegetable intake: had ≥ 1.5 bowls of vegetables every day; Sufficient fruit intake: had ≥ 2 servings of fruit every day; Sufficient sleep: sleep at least 8 h; MTS‐A: Mental Toughness Scale for Adolescents, the scale [18–72] is composed of six dimensions, including challenge [3–12], commitment [3–12], emotion control [3–12], life control [3–12], confidence in abilities [3–12], and interpersonal confidence [3–12], with a higher score indicating higher mental toughness. Based on the sample median, mental toughness was dichotomized into low (≤ 46) and high (> 46); Health literacy level: scores for evaluating personal competencies for the access to, understanding of, appraisal of and application of health information in order to make sound decisions in everyday life. Based on the sample median, health literacy was categorized into low (44–144) and high (145–220). Hosmer‐Lemeshow goodness of fit (GOF) test: *χ* ^2^ = 9.3137, df = 8, *p* = 0.3165. ORs/aORs > 1 indicate that the target group has higher odds of the outcome (physical inactivity) compared with the reference group, whereas ORs/aORs < 1 indicate lower odds. Statistically significant associations (*p* < 0.05) are shown in bold.

Abbreviations: aOR, adjusted odds ratio; CI, confidence interval; OR, odds ratio; SES, socioeconomic status.

Similar findings were observed after adjustment for potential confounders (Table 2). Multivariable logistic regression showed that adolescents aged > 14 years had higher odds of physical inactivity than those aged < 13 years (aOR = 1.627, 95% CI: 1.068–2.478, *p* = 0.023). Excessive screen time on electronic games was also associated with higher odds of physical inactivity (aOR = 1.884, 95% CI: 1.309–2.711, *p* = 0.001). Compared with females, males had lower odds of physical inactivity (aOR = 0.284, 95% CI: 0.198–0.407, *p* < 0.001). Sufficient vegetable intake (aOR = 0.599, 95% CI: 0.389–0.921, *p* = 0.020), excessive screen time on social media (aOR = 0.510, 95% CI: 0.353–0.736, *p* < 0.001), and a higher level of mental toughness (aOR = 0.643, 95% CI: 0.451–0.915, *p* = 0.014) were all associated with lower odds of physical inactivity. These inverse associations remained in the multivariable model for smoking (aOR = 0.351, 95% CI: 0.125–0.988, *p* = 0.047) and alcohol consumption (aOR = 0.507, 95% CI: 0.293–0.877, *p* = 0.015). No significant multicollinearity was observed among the independent variables (Supporting Information S1: Table 2).

### 3.3 Sensitivity Analysis

Sensitivity analyses generally supported the robustness of the main findings. First, analyses using continuous measures of MTS‑A and health literacy yielded results broadly consistent with those of the dichotomized models, suggesting that the main associations were not materially influenced by variable categorization (Supporting Information S1: Table 3). Second, sensitivity analyses using multilevel logistic regression with school included as a random intercept yielded largely consistent findings, although the association with age was attenuated after accounting for school‐level clustering. The intraclass correlation coefficient was 0.101 in the null model and decreased to approximately 0.05 after adjustment for covariates, which falls within the range reported for adolescent health outcomes in secondary schools (Supporting Information S1: Table 4) [44]. Finally, multivariable logistic regression models stratified by gender showed patterns largely consistent with the primary analysis, although some differences in effect sizes and statistical significance were observed between males and females (Supporting Information S1: Tables 5 and 6). Detailed results of these sensitivity analyses are provided in Supporting Information S2: Appendix 2.

## 4 Discussion

### 4.1 Major Findings

In this study, a high proportion of physical inactivity (87.1%) was observed among Hong Kong adolescents. Adolescents aged > 14 years had higher odds of physical inactivity compared with those aged < 13 years. Excessive screen time on electronic games was also associated with higher odds of physical inactivity. In contrast, males had substantially lower odds of being physically inactive than females. Sufficient vegetable intake, excessive social media screen time, and higher mental toughness were also linked to lower odds of inactivity. Additionally, adolescents who reported smoking or alcohol use showed lower odds of physical inactivity compared with their nonsmoking and nondrinking peers, although the prevalence of these behaviors was low.

### 4.2 Comparisons With Existing Studies

Our study revealed a high proportion of physical inactivity among adolescents in Hong Kong, with 87.1% not meeting recommended activity levels. This finding is broadly consistent with global patterns, where adolescent physical inactivity remains widespread. A pooled analysis of 298 cross‐sectional surveys from 146 countries, territories, and areas reported that approximately 81% of adolescents aged 11–17 years were insufficiently active [3]. However, cross‐country comparisons should be interpreted cautiously, as studies differed in measurement tools, age definitions, and cultural and educational contexts, which may contribute to disparity in prevalence estimates [45]. Physical activity in all included studies was assessed using self‑reported measures, consistent with the measurement adopted in our study, typically based on a previously developed instrument [46], in which respondents were asked to report the number of days per week they engaged in at least 60 min of physical activity across all domains. Studies also differed in how adolescence was defined. For example, a repeated cross‐sectional analysis in the United States involving 115,926 adolescents over a 16‐year period found that only 22.8% of adolescents aged 14–17 years reported engaging in more than 60 min of MVPA on all 7 days of the week, while 18.8% reported not achieving this level of activity on any day [47]. Such differences in age range or school grade definitions may partly account for variation in reported prevalence estimates. Beyond these methodological differences, cross‑national differences in adolescent physical inactivity may also reflect cultural and educational contexts. A cross‐sectional study of 347,935 adolescents from 52 countries found substantial cross‐national differences in physical activity, which may partly reflect differences in educational policies, such as differences in PE curriculum time allocation in secondary schools, as well as cultural factors, such as societal beliefs about the importance of physical activity for health and personal or collective identity [13].

In relation to this issue, our study identified several key factors associated with physical inactivity. First, a significant age‐related gradient in physical activity was observed, with adolescents aged > 14 years demonstrating approximately 60% higher odds of physical inactivity relative to those aged < 13 years. Mirroring this trend, prior research tracking physical activity patterns found that daily MVPA duration dropped from approximately 180 min at age 9 to just 49 min (weekdays) and 35 min (weekends) by age 15 [48]. A study of 1110 adolescents in the United States also reported an inverse association between age and physical activity levels [49].

Second, consistent with prior research, males had 72% lower odds of physical inactivity than females, indicating a gender disparity in physical inactivity [3, 36]. This gender disparity is often attributed to broader sociocultural and structural barriers faced by females, such as inequalities in opportunities and social norms restricting female participation in physical activity [50]. Moreover, a global ecological study based on 2016 WHO country‐level data in adults found that countries with greater income inequality tend to exhibit larger gender gaps in physical activity, regardless of their wealth or healthcare spending, suggesting that socioeconomic disparities may contribute to gender differences in physical activity [51].

Vegetable intake was another factor found to be significantly associated with physical activity level, with insufficient consumption being associated with higher physical inactivity. The positive association between vegetable intake and physical activity was also reported in previous studies [37, 38, 52]. A cross‐sectional study of 2057 adolescents from Brazil found that insufficient vegetable intake was associated with a 50% higher likelihood of having inadequate physical activity than those with adequate vegetable intake [37]. The co‐occurrence of physical inactivity and inadequate vegetable consumption has been commonly reported in previous studies [53, 54, 55]. The ecological model of health promotion can help explain this association by examining how personal, interpersonal, and community factors shape individual lifestyle behaviors [56, 57]. For example, adolescents with limited health literacy or living in underserved areas may have reduced access to healthy foods and safe spaces for exercise, promoting both poor diet and inactivity [56, 57].

The finding that adolescents with excessive social media screen time had lower odds of physical inactivity contrasts with some previous studies. While some research found a weak positive association between social media use and sedentary behavior [58], others highlighted social media's potential to promote physical activity by increasing access to health information and motivation through peer influence [59]. Furthermore, exposure to idealized body images on social media may be associated with some adolescents' motivation to increase physical activity to achieve desired physiques [60, 61, 62]. However, this inverse association should be interpreted cautiously. It may reflect residual confounding, as adolescents who are more embedded in active peer networks may exhibit both higher social media engagement and greater physical activity participation, given that peer‐related social connectivity has been shown to correlate with youth physical activity patterns [63]. Therefore, the relationship between social media use and physical activity remains complex and warrants further investigation. In contrast, excessive screen time on electronic gaming was associated with higher odds of physical inactivity in our study, consistent with existing evidence. A questionnaire‐based study of esports players revealed that they engaged in lower levels of physical activity compared to non‐esports players [64]. This sedentary behavior was associated with lower lean body mass and reduced bone mineral content, highlighting substantial negative health implications [64]. A systematic review further supported this association, suggesting that increased screen time on electronic games was linked to adverse lifestyle outcomes in youth, including reduced physical activity levels, elevated body mass index (BMI), and more sedentary behavior [65].

Moreover, our study found that higher levels of mental toughness were associated with lower odds of physical inactivity, which is consistent with prior research [39, 40, 66]. For instance, an online survey of 1426 adolescents reported associations indicating that higher levels of physical activity were linked with greater self‑efficacy and mental toughness, which in turn were associated with lower levels of negative emotions [67]. Sports participation has been described in previous literature as being associated with immediate mood benefits and stress relief, and as relating to the development of resilience skills that may support adolescents in coping with future psychological challenges [68].

### 4.3 Implications for Practice and Research

This study highlights an urgent need for multilevel strategies to address the high proportion of physical inactivity among Hong Kong adolescents. Given the key factors identified such as sex, age, screen time behavior, dietary habits, and mental toughness, tailored strategies may be useful in future school‐ and community‐based physical activity promotion. School‐based programs may particularly prioritize girls and older students, who showed higher odds of inactivity in this study. Potential strategies include integrating structured exercise into the curriculum, expanding access to extracurricular sports, and promoting inclusive, gender‐sensitive environments. At the community level, digital platforms may be used to deliver physical activity messages, while future programs should distinguish between different types of screen use, particularly electronic gaming and social media. Community centers and youth programs may also offer accessible, appealing physical activity options that promote psychological resilience. Mental toughness was associated with adolescents' physical activity patterns in our study, suggesting that future initiatives may consider incorporating components that support psychological attributes such as confidence, persistence, and self‐regulation when promoting active lifestyles. Given the observed associations with dietary behaviors, integrating nutrition education with physical activity initiatives may be a practical component within a broader healthy lifestyle framework.

Future research should focus on designing and evaluating interventions that combine physical activity promotion with improvements in mental toughness, dietary behaviors, and digital literacy. Longitudinal studies are also needed to clarify the observed clustering of behaviors, including the associations involving media use, smoking, alcohol use, and physical activity, to better guide holistic adolescent health strategies.

### 4.4 Strengths and Limitations

This study has several strengths. It utilized validated instruments to assess mental toughness and behavioral factors, enhancing measurement reliability. The inclusion of a broad range of covariates allowed for more accurate adjustment of potential confounders. Additionally, the large sample size improved the statistical power and reliability of the findings.

Nonetheless, several limitations should be acknowledged. First, the use of convenience sampling of secondary schools participating in the “Health Education Built‐on Project” may introduce selection bias and limit generalizability. Although participating schools were located across 11 of the 18 clusters in Hong Kong, only 20 of 440 invited schools participated, and no data were available to compare participating and nonparticipating schools. Therefore, the magnitude and direction of potential selection bias cannot be determined. Second, physical activity‐related behaviors were assessed using a single self‑reported item, which may introduce recall bias and misclassification, particularly among younger participants such as those aged below 13 years, who may have limited ability to accurately estimate daily moderate‑to‑vigorous activity or other health behaviors. However, this item was adapted from the WHO GSHS and has demonstrated acceptable reliability in adolescent populations across diverse settings, supporting its use in large‑scale epidemiologic research despite these limitations [18, 19, 20, 21]. Third, the interpretation of the inverse associations involving social media, smoking, and alcohol use should be approached with caution. The estimates for smoking and alcohol use were based on relatively small numbers of participants reporting these behaviors, while the association with social media use may be influenced by unmeasured differences in peer networks, activity interests, or patterns of social media engagement. Residual confounding, reverse causation, and measurement limitations may therefore have contributed to these findings. Given the cross‐sectional design, these results reflect associations rather than causal relationships and should be confirmed in future longitudinal studies and studies using more representative samples. Additionally, obesity was assessed using self‐reported status rather than objective measurements such as BMI calculated from height and weight. Self‐reported measures may be subject to misclassification bias, as participants may underreport or overreport their obesity status. This could lead to potential attenuation of the observed associations. Future studies should incorporate objective anthropometric measurements to improve accuracy. Finally, as the study focused solely on Hong Kong adolescents, findings may not be generalizable to other contexts. Future longitudinal and cross‐regional studies are warranted to confirm and extend these results.

## 5 Conclusion

This study found a concerningly high proportion of physical inactivity among surveyed adolescents in Hong Kong and identified key demographic, behavioral, and psychosocial factors associated with inactivity. These findings may inform targeted, multilevel strategies in school and community settings to promote active lifestyles among adolescents. Future longitudinal and intervention studies are needed to determine how these associated factors can be addressed to improve adolescent physical activity and long‐term health outcomes.

## Author Contributions

**Claire Chenwen Zhong:** conceptualization, supervision, writing – original draft. **Zehuan Yang:** writing – original draft. **Mingtao Chen:** writing – original draft. **Zhaojun Li:** data curation, formal analysis. **Vera M. W. Keung:** data curation, formal analysis. **Amelia Lo:** data curation, formal analysis. **Calvin Cheung:** data curation, formal analysis. **Junjie Huang:** conceptualization, supervision, writing – review and editing. **Martin C. S. Wong:** conceptualization, supervision, writing – review and editing. All authors read and approved the final manuscript.

## Disclosure

Prof Martin C. S. Wong affirms that this manuscript is an honest, accurate, and transparent account of the study being reported; that no important aspects of the study have been omitted; and that any discrepancies from the study as planned (and, if relevant, registered) have been explained.

## Ethics Statement

The Survey and Behavioural Research Ethics Committee of the Chinese University of Hong Kong approved the study (Approval No. SBRE‐24‐0029).

## Consent

School consent was obtained through a project application form. Informed consent to participate in the study was obtained from all students, and from their parents or legal guardians for participants under 16. Two templates of parent consent forms were provided to participating schools, allowing them to select either an opt‐in or opt‐out approach for collecting parental consent based on their specific circumstances.

## Conflicts of Interest

M.C.S.W. is an honorary medical advisor of GenieBiome Ltd., SunRise, and BGI Health. He is an advisory committee member of Pfizer; an external expert of GlaxoSmithKline Limited; a member of the advisory board of AstraZeneca, and has been paid consultancy fees for providing advice on research. He has received grants from Pfizer, Merck Sharp & Dohme, AstraZeneca, GSK, and Moderna. These grants were not used to support the conduct, analysis, or reporting of the present study. The other authors declared no potential conflicts of interest with respect to the research, authorship, and/or publication of this article.

## Declaration of generative AI and AI‐assisted technologies in the writing process

During the preparation of this work, the authors used ChatGPT only to improve language and readability, with caution. After using this tool, the authors reviewed and edited the content as needed and took full responsibility for the content of the publication.

## Supporting information


Supporting File 1

Supporting File 2

## Data Availability

The data sets used and/or analyzed during the current study are available from the corresponding author on reasonable request. Prof Martin C. S. Wong had full access to all of the data in this study and takes complete responsibility for the integrity of the data and the accuracy of the data analysis.

## References

[1] World Health Organization , *WHO Guidelines on Physical Activity and Sedentary Behaviour* (WHO, 2020), https://iris.who.int/bitstream/handle/10665/336656/9789240015128‐eng.pdf?sequence=1. 33369898

[2] T. Strain , S. Flaxman , R. Guthold , et al., “National, Regional, and Global Trends in Insufficient Physical Activity Among Adults From 2000 to 2022: A Pooled Analysis of 507 Population‐Based Surveys With 5.7 Million Participants,” Lancet Global Health 12, no. 8 (2024): e1232–e1243. 38942042 10.1016/S2214-109X(24)00150-5 PMC11254784

[3] R. Guthold , G. A. Stevens , L. M. Riley , and F. C. Bull , “Global Trends in Insufficient Physical Activity Among Adolescents: A Pooled Analysis of 298 Population‐Based Surveys With 1.6 Million Participants,” Lancet Child & Adolescent Health 4, no. 1 (2020): 23–35. 31761562 10.1016/S2352-4642(19)30323-2 PMC6919336

[4] P. N. Kemel , J. E. Porter , and N. Coombs , “Improving Youth Physical, Mental and Social Health Through Physical Activity: A Systematic Literature Review,” Health Promotion Journal of Australia 33, no. 3 (2022): 590–601. 34735738 10.1002/hpja.553

[5] L. Redig , N. Feter , S. C. Dumith , M. R. Domingues , and A. J. Rombaldi , “Physical Inactivity From Childhood to Adolescence and Incident Depression,” American Journal of Preventive Medicine 62, no. 2 (2022): 211–218. 34702605 10.1016/j.amepre.2021.09.001

[6] P. Kallio , K. Pahkala , O. J. Heinonen , et al., “Physical Inactivity From Youth to Adulthood and Adult Cardiometabolic Risk Profile,” Preventive Medicine 145 (2021): 106433. 33497685 10.1016/j.ypmed.2021.106433

[7] M. Hirvensalo and T. Lintunen , “Life‐Course Perspective for Physical Activity and Sports Participation,” European Review of Aging and Physical Activity 8 (2011): 13–22.

[8] E. M. F. van Sluijs , U. Ekelund , I. Crochemore‐Silva , et al., “Physical Activity Behaviours in Adolescence: Current Evidence and Opportunities for Intervention,” Lancet 398, no. 10298 (2021): 429–442. 34302767 10.1016/S0140-6736(21)01259-9 PMC7612669

[9] E. S. Cowley , P. M. Watson , L. Foweather , et al., “‘Girls Aren't Meant to Exercise’: Perceived Influences on Physical Activity Among Adolescent Girls—The HERizon Project,” Children 8, no. 1 (2021): 31. 33430413 10.3390/children8010031 PMC7827342

[10] P. W. Henriksen , S. B. Rayce , O. Melkevik , P. Due , and B. E. Holstein , “Social Background, Bullying, and Physical Inactivity: National Study of 11‐ to 15‐Year‐Olds,” Scandinavian Journal of Medicine & Science in Sports 26, no. 10 (2016): 1249–1255. 26454139 10.1111/sms.12574

[11] O. Heradstveit , S. Haugland , M. Hysing , K. M. Stormark , B. Sivertsen , and T. Bøe , “Physical Inactivity, Non‐Participation in Sports and Socioeconomic Status: A Large Population‐Based Study Among Norwegian Adolescents,” BMC Public Health 20 (2020): 1010. 32590961 10.1186/s12889-020-09141-2 PMC7318733

[12] W. Y. Huang , S. H. S. Wong , C. H. P. Sit , M. C. S. Wong , S. W. S. Wong , and R. S. T. Ho , “Results From the Hong Kong's 2022 Report Card on Physical Activity for Children and Adolescents,” Journal of Exercise Science and Fitness 21, no. 1 (2023): 45–51. 36408208 10.1016/j.jesf.2022.10.010 PMC9649951

[13] D. Bann , S. Scholes , M. Fluharty , and N. Shure , “Adolescents' Physical Activity: Cross‐National Comparisons of Levels, Distributions and Disparities Across 52 Countries,” International Journal of Behavioral Nutrition and Physical Activity 16, no. 1 (2019): 141. 31888652 10.1186/s12966-019-0897-z PMC6937925

[14] A. Carver , M. Akram , A. Barnett , et al., “Family, School and Individual Characteristics Associated With Adolescents' Physical Activity at School in Hong Kong: The iHealt(H) Study,” International Journal of Behavioral Nutrition and Physical Activity 18, no. 1 (2021): 14. 33468170 10.1186/s12966-021-01085-z PMC7816388

[15] E. Von Elm , D. G. Altman , M. Egger , S. J. Pocock , P. C. Gøtzsche , and J. P. Vandenbroucke , “The Strengthening the Reporting of Observational Studies in Epidemiology (STROBE) Statement: Guidelines for Reporting Observational Studies,” Lancet 370, no. 9596 (2007): 1453–1457. 18064739 10.1016/S0140-6736(07)61602-X

[16] Education Bureau , *Student Enrolment Statistics, 2024/25 (Kindergartens, Primary and Secondary Schools)* (Education Bureau, 2025), https://www.edb.gov.hk/attachment/en/about‐edb/publications‐stat/figures/Enrol_2024.pdf.

[17] World Health Organization (WHO) , “GSHS Questionnaire,” WHO, https://www.who.int/teams/noncommunicable‐diseases/surveillance/systems‐tools/global‐school‐based‐student‐health‐survey/questionnaire.

[18] K. S. Starks , D. Kamara , and K. H. Jacobsen , “Sedentary Behavior and Physical Inactivity Among Secondary School Students in the 2017 Sierra Leone Global School‐Based Student Health Survey,” Journal of School Health 94, no. 5 (2024): 433–442. 37883953 10.1111/josh.13402

[19] M. H. Alkhraiji , A. R. Barker , and C. A. Williams , “Reliability and Validity of Using the Global School‐Based Student Health Survey to Assess 24 Hour Movement Behaviours in Adolescents From Saudi Arabia,” Journal of Sports Sciences 40, no. 14 (2022): 1578–1586. 35762915 10.1080/02640414.2022.2092982

[20] G. Xu , N. Sun , L. Li , et al., “Physical Behaviors of 12‐15 Year‐Old Adolescents in 54 Low‐ and Middle‐Income Countries: Results From the Global School‐Based Student Health Survey,” Journal of Global Health 10, no. 1 (2020): 010423. 32426123 10.7189/jogh.10.010423 PMC7211419

[21] R. Guthold , M. J. Cowan , C. S. Autenrieth , L. Kann , and L. M. Riley , “Physical Activity and Sedentary Behavior Among Schoolchildren: A 34‐Country Comparison,” Journal of Pediatrics 157, no. 1 (2010): 43–49.e1. 20304415 10.1016/j.jpeds.2010.01.019

[22] Y. Liu , M. Wang , J. Villberg , et al., “Reliability and Validity of Family Affluence Scale (FAS II) Among Adolescents in Beijing, China,” Child Indicators Research 5 (2012): 235–251.

[23] S. Ghanbari , A. Ramezankhani , A. Montazeri , and Y. Mehrabi , “Health Literacy Measure for Adolescents (HELMA): Development and Psychometric Properties,” PLoS One 11, no. 2 (2016): e0149202. 26881933 10.1371/journal.pone.0149202 PMC4755574

[24] S. A. Fleary , P. Joseph , and J. E. Pappagianopoulos , “Adolescent Health Literacy and Health Behaviors: A Systematic Review,” Journal of Adolescence 62 (2018): 116–127. 29179126 10.1016/j.adolescence.2017.11.010

[25] Centre for Health Protection , *Healthy Eating Food Pyramid for Adolescents 12–17 Years Old* (Centre for Health Protection, 2024), https://www.chp.gov.hk/files/her/exn_nutp_029bp.pdf.

[26] M. S. Tremblay , V. Carson , J. P. Chaput , et al., “Canadian 24‐Hour Movement Guidelines for Children and Youth: An Integration of Physical Activity, Sedentary Behaviour, and Sleep,” Applied Physiology, Nutrition, and Metabolism 41, no. 6 Suppl. 3 (2016): S311–S327. 10.1139/apnm-2016-0151 27306437

[27] National Heart, Lung, and Blood Institute , How Much Sleep Is Enough (National Heart, Lung, and Blood Institute, 2022), https://www.nhlbi.nih.gov/health/sleep/how‐much‐sleep.

[28] S. McGeown , H. St. Clair‐Thompson , and D. W. Putwain , “The Development and Validation of a Mental Toughness Scale for Adolescents,” Journal of Psychoeducational Assessment 36, no. 2 (2018): 148–161.

[29] J. DeCoster , A. M. R. Iselin , and M. Gallucci , “A Conceptual and Empirical Examination of Justifications for Dichotomization,” Psychological Methods 14, no. 4 (2009): 349–366. 19968397 10.1037/a0016956

[30] D. G. Altman and P. Royston , “The Cost of Dichotomising Continuous Variables,” BMJ 332, no. 7549 (2006): 1080. 16675816 10.1136/bmj.332.7549.1080 PMC1458573

[31] J. DeCoster , M. Gallucci , and A.‐M. R. Iselin , “Best Practices for Using Median Splits, Artificial Categorization, and Their Continuous Alternatives,” Journal of Experimental Psychopathology 2, no. 2 (2011): 197–209.

[32] M. Assel , D. Sjoberg , A. Elders , et al., “Guidelines for Reporting of Statistics for Clinical Research in Urology,” BJU International 123, no. 3 (2019): 401–410. 30537407 10.1111/bju.14640 PMC6397060

[33] D. W. Hosmer Jr., S. Lemeshow , and R. X. Sturdivant , Applied Logistic Regression (John Wiley & Sons, 2013).

[34] S. Greenland , “Modeling and Variable Selection in Epidemiologic Analysis,” American Journal of Public Health 79, no. 3 (1989): 340–349. 2916724 10.2105/ajph.79.3.340 PMC1349563

[35] Z. Bursac , C. H. Gauss , D. K. Williams , and D. W. Hosmer , “Purposeful Selection of Variables in Logistic Regression,” Source Code for Biology and Medicine 3 (2008): 17. 19087314 10.1186/1751-0473-3-17 PMC2633005

[36] G. I. Mielke , I. C. M. da Silva , T. L. Kolbe‐Alexander , and W. J. Brown , “Shifting the Physical Inactivity Curve Worldwide by Closing the Gender Gap,” Sports Medicine 48, no. 2 (2018): 481–489. 28647914 10.1007/s40279-017-0754-7

[37] D. A. Silva and R. J. Silva , “Association Between Physical Activity Level and Consumption of Fruit and Vegetables Among Adolescents in Northeast Brazil,” Revista Paulista de Pediatria 33, no. 2 (2015): 167–173. 25887930 10.1016/j.rpped.2014.09.003 PMC4516370

[38] S. A. Darfour‐Oduro , J. E. Andrade , and D. S. Grigsby‐Toussaint , “Do Fruit and Vegetable Policies, Socio‐Environmental Factors, and Physical Activity Influence Fruit and Vegetable Intake Among Adolescents?,” Journal of Adolescent Health 66, no. 2 (2020): 172–180. 10.1016/j.jadohealth.2019.07.016 31564617

[39] G. E. Hale , L. Colquhoun , D. Lancastle , N. Lewis , and P. J. Tyson , “Review: Physical Activity Interventions for the Mental Health and Well‐Being of Adolescents – A Systematic Review,” Child and Adolescent Mental Health 26, no. 4 (2021): 357–368. 34105239 10.1111/camh.12485

[40] T. M. Wassenaar , C. M. Wheatley , N. Beale , et al., “The Effect of a One‐Year Vigorous Physical Activity Intervention on Fitness, Cognitive Performance and Mental Health in Young Adolescents: The Fit to Study Cluster Randomised Controlled Trial,” International Journal of Behavioral Nutrition and Physical Activity 18, no. 1 (2021): 47. 33789683 10.1186/s12966-021-01113-y PMC8011147

[41] J. Huang , C. K. Cheung , S. C. Chan , et al., “Factors Associated With Physical Inactivity Among the Pre‐School Children: A Cohort of 1681 Participants,” Journal of Paediatrics and Child Health 59, no. 10 (2023): 1152–1159. 37574970 10.1111/jpc.16473

[42] L. C. G. Martins , M. V. O. Lopes , C. M. Diniz , and N. G. Guedes , “The Factors Related to a Sedentary Lifestyle: A Meta‐Analysis Review,” Journal of Advanced Nursing 77, no. 3 (2021): 1188–1205. 33368524 10.1111/jan.14669

[43] M. H. Kutner , Applied Linear Statistical Models (McGraw‐Hill Irwin, 2005).

[44] N. Shackleton , D. Hale , C. Bonell , and R. M. Viner , “Intraclass Correlation Values for Adolescent Health Outcomes in Secondary Schools in 21 European Countries,” SSM – Population Health 2 (2016): 217–225. 29349141 10.1016/j.ssmph.2016.03.005 PMC5757888

[45] S. Aubert , J. Brazo‐Sayavera , S. A. González , et al., “Global Prevalence of Physical Activity for Children and Adolescents; Inconsistencies, Research Gaps, and Recommendations: A Narrative Review,” International Journal of Behavioral Nutrition and Physical Activity 18, no. 1 (2021): 81. 34187486 10.1186/s12966-021-01155-2 PMC8243483

[46] J. J. Prochaska , J. F. Sallis , and B. Long , “A Physical Activity Screening Measure for Use With Adolescents in Primary Care,” Archives of Pediatrics & Adolescent Medicine 155, no. 5 (2001): 554–559. 11343497 10.1001/archpedi.155.5.554

[47] S. Chen , D. Brown , C. D. Pfledderer , W. Y. Huang , and M. S. Tremblay , “Temporal Trends of No Moderate to Vigorous Physical Activity in Adolescents: A 16‐Year Trend Analysis of 115,926 Participants,” International Journal of Behavioral Nutrition and Physical Activity 23, no. 1 (2025): 3. 41345642 10.1186/s12966-025-01862-0 PMC12797897

[48] P. R. Nader , R. H. Bradley , R. M. Houts , S. L. McRitchie , and M. O'Brien , “Moderate‐to‐Vigorous Physical Activity From Ages 9 to 15 Years,” Journal of the American Medical Association 300, no. 3 (2008): 295–305. 18632544 10.1001/jama.300.3.295

[49] S. G. Trost , R. R. Pate , J. F. Sallis , et al., “Age and Gender Differences in Objectively Measured Physical Activity in Youth,” Medicine and Science in Sports and Exercise 34, no. 2 (2002): 350–355. 11828247 10.1097/00005768-200202000-00025

[50] A. Moreno‐Llamas , J. García‐Mayor , and E. De la Cruz‐Sánchez , “Gender Inequality Is Associated With Gender Differences and Women Participation in Physical Activity,” Journal of Public Health 44, no. 4 (2021): e519–e526. 10.1093/pubmed/fdab354 34604911

[51] C. Sfm , J. Van Cauwenberg , L. Maenhout , G. Cardon , E. V. Lambert , and D. Van Dyck , “Inequality in Physical Activity, Global Trends by Income Inequality and Gender in Adults,” International Journal of Behavioral Nutrition and Physical Activity 17, no. 1 (2020): 142. 33239036 10.1186/s12966-020-01039-x PMC7690175

[52] C. T. Ndagire , J. H. Muyonga , and D. Nakimbugwe , “Fruit and Vegetable Consumption, Leisure‐Time Physical Activity, and Sedentary Behavior Among Children and Adolescent Students in Uganda,” Food Science & Nutrition 7, no. 2 (2019): 599–607. 30847139 10.1002/fsn3.883 PMC6392852

[53] S. P. J. Kremers , D. B. Gert‐Jan , S. Herman , and J. Brug , “Clustering of Energy Balance‐Related Behaviours and Their Intrapersonal Determinants,” Psychology & Health 19, no. 5 (2004): 595–606.

[54] A. Sanchez , G. J. Norman , J. F. Sallis , K. J. Calfas , J. Cella , and K. Patrick , “Patterns and Correlates of Physical Activity and Nutrition Behaviors in Adolescents,” American Journal of Preventive Medicine 32, no. 2 (2007): 124–130. 17197153 10.1016/j.amepre.2006.10.012 PMC1913476

[55] N. Pearson , A. J. Atkin , S. J. Biddle , T. Gorely , and C. Edwardson , “Patterns of Adolescent Physical Activity and Dietary Behaviours,” International Journal of Behavioral Nutrition and Physical Activity 6, no. 1 (2009): 45. 19624822 10.1186/1479-5868-6-45 PMC2718856

[56] S. Kelly , B. M. Melnyk , and M. Belyea , “Predicting Physical Activity and Fruit and Vegetable Intake in Adolescents: A Test of the Information, Motivation, Behavioral Skills Model,” Research in Nursing & Health 35, no. 2 (2012): 146–163. 22262049 10.1002/nur.21462

[57] D. A. S. Silva , K. G. Peres , A. F. Boing , D. A. González‐Chica , and M. A. Peres , “Clustering of Risk Behaviors for Chronic Noncommunicable Diseases: A Population‐Based Study in Southern Brazil,” Preventive Medicine 56, no. 1 (2013): 20–24. 23123860 10.1016/j.ypmed.2012.10.022

[58] S. V. Shimoga , E. Erlyana , and V. Rebello , “Associations of Social Media Use With Physical Activity and Sleep Adequacy Among Adolescents: Cross‐Sectional Survey,” Journal of Medical Internet Research 21, no. 6 (2019): e14290. 31215512 10.2196/14290 PMC6604510

[59] V. A. Goodyear , G. Wood , B. Skinner , and J. L. Thompson , “The Effect of Social Media Interventions on Physical Activity and Dietary Behaviours in Young People and Adults: A Systematic Review,” International Journal of Behavioral Nutrition and Physical Activity 18, no. 1 (2021): 72. 34090469 10.1186/s12966-021-01138-3 PMC8180076

[60] G. Holland and M. Tiggemann , “A Systematic Review of the Impact of the Use of Social Networking Sites on Body Image and Disordered Eating Outcomes,” Body Image 17 (2016): 100–110. 26995158 10.1016/j.bodyim.2016.02.008

[61] A. Keyes , S. Woerwag‐Mehta , S. Bartholdy , et al., “Physical Activity and the Drive to Exercise in Anorexia Nervosa,” International Journal of Eating Disorders 48, no. 1 (2015): 46–54. 25196139 10.1002/eat.22354

[62] R. Melissa , M. Lama , K. Laurence , et al., “Physical Activity in Eating Disorders: A Systematic Review,” Nutrients 12, no. 1 (2020): 183. 31936525 10.3390/nu12010183 PMC7019575

[63] S. Levi and O. Baron‐Epel , “Adolescent Perspectives on the Impact of Peers and Social Media on Active Travel and Physical Activity: A Mixed Methods Study,” Journal of Adolescent Research 40, no. 2 (2025): 512–543.

[64] J. DiFrancisco‐Donoghue , W. G. Werner , P. C. Douris , and H. Zwibel , “Esports Players, Got Muscle? Competitive Video Game Players' Physical Activity, Body Fat, Bone Mineral Content, and Muscle Mass in Comparison to Matched Controls,” Journal of Sport and Health Science 11, no. 6 (2022): 725–730. 32711155 10.1016/j.jshs.2020.07.006 PMC9729923

[65] G. Chan , Y. Huo , S. Kelly , J. Leung , C. Tisdale , and M. Gullo , “The Impact of eSports and Online Video Gaming on Lifestyle Behaviours in Youth: A Systematic Review,” Computers in Human Behavior 126 (2022): 106974.

[66] A. Khan , E.‐Y. Lee , S. Rosenbaum , S. R. Khan , and M. S. Tremblay , “Dose‐Dependent and Joint Associations Between Screen Time, Physical Activity, and Mental Wellbeing in Adolescents: An International Observational Study,” Lancet Child & Adolescent Health 5, no. 10 (2021): 729–738. 34384543 10.1016/S2352-4642(21)00200-5

[67] H. Yu and Q. Mu , “Effect of Physical Exercise on Negative Emotions in Adolescent: A Chain‐Mediated Effect of Self‐efficacy and Mental Toughness,” preprint, Research Square, 2023.

[68] A. Martín‐Rodríguez , L. A. Gostian‐Ropotin , A. I. Beltrán‐Velasco , et al., “Sporting Mind: The Interplay of Physical Activity and Psychological Health,” Sports 12, no. 1 (2024): 37. 38275986 10.3390/sports12010037 PMC10819297

