# Supplementary material for: Exploring Patterns and Associated Factors of Physical Inactivity in Adolescents: A Cross‑Sectional Study
Source: Health Sci Rep. 2026 Aug 19;9(8):e72926. doi: 10.1002/hsr2.72926 (PMC13490853; doi:10.1002/hsr2.72926)
Supplement: Supplementary file 1 [file HSR2-9-e72926-s001.docx]

**Supplementary Material**

Supplementary Table 1.	Descriptive statistics of continuous variables

Supplementary Table 2.	Multicollinearity detection results

Supplementary Table 3.	Factors associated with insufficient physical activity frequency using continuous measures of MTS-A and health literacy

Supplementary Table 4.	Multilevel logistic regression analysis accounting for school-level clustering

Supplementary Table 5.	Factors associated with physical inactivity frequency in male group

Supplementary Table 6.	Factors associated with physical inactivity frequency in female group

Supplementary Figure 1.	Q-Q plot for continuous variables

Supplementary Table 1. Descriptive statistics of continuous variables

| **Variable** | **Mean** | **SD** | **Median** | **IQR** | **Min** | **Max** | **Skewness** | **Kurtosis** |
| --- | --- | --- | --- | --- | --- | --- | --- | --- |
| MTS | 46.19 | 8.04 | 46 | 10 | 21 | 72 | 0.15 | 3.56 |
| Health literacy level | 144.74 | 29.99 | 144 | 39 | 44 | 220 | -0.27 | 3.47 |

MTS-A: Mental Toughness Scale for Adolescents; SD, standard deviation; IQR, interquartile range.

Supplementary Table 2. Multicollinearity detection results

|  | **GVIF** | **Df** | **GVIF^(1/(2*Df))** |
| --- | --- | --- | --- |
| Age group | 1.132634 | 2 | 1.031626 |
| Gender | 1.13851 | 1 | 1.06701 |
| Smoking | 1.099239 | 1 | 1.048446 |
| Alcohol drinking | 1.137174 | 1 | 1.066384 |
| SES | 1.070544 | 2 | 1.017188 |
| Having breakfast 7 days in a week | 1.088421 | 1 | 1.043274 |
| Sufficient vegetable intake | 1.065549 | 1 | 1.032255 |
| Sufficient fruit intake | 1.089318 | 1 | 1.043704 |
| Sufficient sleep | 1.110706 | 1 | 1.0539 |
| Excessive screen time on video | 1.176495 | 1 | 1.084663 |
| Excessive screen time on electronic game | 1.242573 | 1 | 1.114708 |
| Excessive screen time on social media | 1.260077 | 1 | 1.122532 |
| Self-reported obesity | 1.022314 | 1 | 1.011096 |
| Health literacy level | 1.142715 | 1 | 1.068979 |
| MTS-A | 1.148554 | 1 | 1.071706 |

Sufficient physical activity: Moderate/Vigorous exercise >=60 minutes every day; Physical inactivity: Moderate/Vigorous exercise <60 minutes every day; SES: Socioeconomic Status; Sufficient vegetable intake: Had ≥ 1.5 bowls of vegetables every day; Sufficient fruit intake: Had ≥ 2 servings of fruit every day; Sufficient sleep: Sleep at least 8 hours; MTS-A: Mental Toughness Scale for Adolescents; Health literacy level: Scores for evaluating personal competencies for the access to, understanding of, appraisal of and application of health information in order to make sound decisions in everyday life.

Supplementary Table 3. Factors associated with insufficient physical activity frequency using continuous measures of MTS-A and health literacy

|  | OR (95% CI)* | *P-value* | aOR (95% CI) | *P-value* |
| --- | --- | --- | --- | --- |
| **Age group** |  |  |  |  |
| <13 | reference |  | reference |  |
| 13-14 | 1.289 (0.888-1.871) | 0.182 | 1.462 (0.977-2.188) | 0.065 |
| >14 | 1.492 (1.016-2.190) | **0.041** | 1.600 (1.050-2.437) | **0.029** |
| **Gender** |  |  |  |  |
| Female | reference |  | reference |  |
| Male | 0.324 (0.233-0.450) | **<0.001** | 0.309 (0.214-0.445) | **<0.001** |
| **SES** |  |  |  |  |
| Low | reference |  | reference |  |
| Medium | 0.783 (0.486-1.263) | 0.317 | 0.835 (0.506-1.380) | 0.483 |
| High | 0.657 (0.402-1.073) | 0.094 | 0.759 (0.448-1.284) | 0.303 |
| **Smoking** |  |  |  |  |
| No | reference |  | reference |  |
| Yes | 0.331 (0.134-0.815) | **0.016** | 0.340 (0.121-0.955) | **0.041** |
| **Alcohol drinking** |  |  |  |  |
| No | reference |  | reference |  |
| Yes | 0.554 (0.343-0.892) | **0.015** | 0.511 (0.295-0.886) | **0.017** |
| **Having breakfast 7 days in a week** |  |  |  |  |
| No | reference |  | reference |  |
| Yes | 0.706 (0.517-0.965) | **0.029** | 0.853 (0.606-1.199) | 0.360 |
| **Sufficient vegetable intake** |  |  |  |  |
| No | reference |  | reference |  |
| Yes | 0.570 (0.384-0.845) | **0.005** | 0.602 (0.390-0.928) | **0.022** |
| **Sufficient fruit intake** |  |  |  |  |
| No | reference |  | reference |  |
| Yes | 0.628 (0.429-0.918) | **0.016** | 0.821 (0.537-1.254) | 0.361 |
| **Sufficient sleep** |  |  |  |  |
| No | reference |  | reference |  |
| Yes | 0.714 (0.524-0.974) | **0.034** | 0.844 (0.599-1.190) | 0.333 |
| **Excessive screen time on video** |  |  |  |  |
| No | reference |  | reference |  |
| Yes | 1.002 (0.732-1.371) | 0.992 | 0.915 (0.639-1.311) | 0.630 |
| **Excessive screen time on electronic game** |  |  |  |  |
| No | reference |  | reference |  |
| Yes | 1.409 (1.032-1.923) | **0.031** | 1.900 (1.319-2.736) | **0.001** |
| **Excessive screen time on social media** |  |  |  |  |
| No | reference |  | reference |  |
| Yes | 0.771 (0.564-1.054) | 0.102 | 0.505 (0.349-0.730) | **<0.001** |
| **Self-reported obesity** |  |  |  |  |
| No | reference |  | reference |  |
| Yes | 1.340 (0.952-1.885) | 0.093 | 1.156 (0.805-1.661) | 0.432 |
|  |  |  |  |  |
| **MTS-A** | 0.942 (0.924-0.961) | **<0.001** | 0.962 (0.940-0.985) | **0.001** |
|  |  |  |  |  |
| **Health literacy level** | 0.995 (0.989-1.000) | **0.042** | 0.998 (0.993-1.004) | 0.612 |

Sufficient physical activity: Moderate/Vigorous exercise >=60 minutes every day; Physical inactivity: Moderate/Vigorous exercise <60 minutes every day; SES: Socioeconomic Status; Sufficient vegetable intake: Had ≥ 1.5 bowls of vegetables every day; Sufficient fruit intake: Had ≥ 2 servings of fruit every day; Sufficient sleep: Sleep at least 8 hours; MTS-A: Mental Toughness Scale for Adolescents; Health literacy level: Scores for evaluating personal competencies for the access to, understanding of, appraisal of and application of health information in order to make sound decisions in everyday life. Significant variables (p<0.05) were in bold.

Hosmer and Lemeshow goodness of fit (GOF) test:

X-squared = 5.8629, df = 8, p-value = 0.6626.

CI: confidence interval; OR, odds ratio; aOR, adjusted odds ratio. ORs / aORs > 1 indicate that the target group has higher odds of the outcome (physical inactivity) compared with the reference group, whereas ORs / aORs <1 indicate lower odds.

Supplementary Table 4. Multilevel logistic regression analysis accounting for school-level clustering

| **Variable** | **aOR (95% CI)** | **P-value** |
| --- | --- | --- |
| **Age group** |  |  |
| <13 | reference |  |
| 13–14 | 1.165 (0.712, 1.906) | 0.543 |
| >14 | 1.189 (0.668, 2.114) | 0.556 |
| **Gender** |  |  |
| Female | reference |  |
| Male | 0.337 (0.229, 0.498) | **<0.001** |
| **Smoking** |  |  |
| No | reference |  |
| Yes | 0.335 (0.117, 0.957) | **0.041** |
| **Alcohol drinking** |  |  |
| No | reference |  |
| Yes | 0.523 (0.3, 0.911) | **0.022** |
| **SES** |  |  |
| Low | reference |  |
| Medium | 0.846 (0.508, 1.407) | 0.519 |
| High | 0.804 (0.469, 1.379) | 0.428 |
| **Having breakfast 7 days/week** |  |  |
| No | reference |  |
| Yes | 0.828 (0.586, 1.17) | 0.285 |
| **Sufficient vegetable intake** |  |  |
| No | reference |  |
| Yes | 0.609 (0.393, 0.944) | **0.026** |
| **Sufficient fruit intake** |  |  |
| No | reference |  |
| Yes | 0.852 (0.553, 1.312) | 0.467 |
| **Sufficient sleep** |  |  |
| No | reference |  |
| Yes | 0.838 (0.591, 1.188) | 0.321 |
| **Excessive screen time (video)** |  |  |
| No | reference |  |
| Yes | 0.934 (0.651, 1.342) | 0.713 |
| **Excessive screen time (game)** |  |  |
| No | reference |  |
| Yes | 1.863 (1.29, 2.691) | **<0.001** |
| **Excessive screen time (social media)** |  |  |
| No | reference |  |
| Yes | 0.521 (0.359, 0.755) | **<0.001** |
| **Self-reported obesity** |  |  |
| No | reference |  |
| Yes | 1.159 (0.805, 1.67) | 0.427 |
| **MTS-A** |  |  |
| Low | reference |  |
| High | 0.641 (0.448, 0.918) | **0.015** |
| **Health literacy level** |  |  |
| Low | reference |  |
| High | 0.745 (0.521, 1.065) | 0.106 |

Multilevel logistic regression model with school included as a random intercept.

Sufficient physical activity: Moderate/Vigorous exercise >=60 minutes every day; Physical inactivity: Moderate/Vigorous exercise <60 minutes every day; SES: Socioeconomic Status; Sufficient vegetable intake: Had ≥ 1.5 bowls of vegetables every day; Sufficient fruit intake: Had ≥ 2 servings of fruit every day; Sufficient sleep: Sleep at least 8 hours; MTS-A: Mental Toughness Scale for Adolescents; Health literacy level: Scores for evaluating personal competencies for the access to, understanding of, appraisal of and application of health information in order to make sound decisions in everyday life. Significant variables (p<0.05) were in bold.

CI: confidence interval; aOR, adjusted odds ratio. aORs > 1 indicate that the target group has higher odds of the outcome (physical inactivity) compared with the reference group,

Model fit: Intraclass correlation coefficient (ICC) = 0.101 for the null model and 0.050 for the adjusted model.

Conditional R^2^: 0.201

Marginal R^2^: 0.159

Supplementary Table 5. Factors associated with physical inactivity frequency in male group

|  | OR (95% CI)* | *P-value* | aOR (95% CI) | *P-value* |
| --- | --- | --- | --- | --- |
| **Age group** |  |  |  |  |
| <13 | reference |  | reference |  |
| 13, 14 | 1.353 (0.851, 2.153) | 0.201 | 1.410 (0.860, 2.309) | 0.173 |
| >14 | 1.329 (0.814, 2.169) | 0.256 | 1.322 (0.774, 2.257) | 0.306 |
| **SES** |  |  |  |  |
| Low | reference |  | reference |  |
| Medium | 0.718 (0.399, 1.293) | 0.270 | 0.749 (0.406, 1.383) | 0.356 |
| High | 0.493 (0.269, 0.905) | **0.022** | 0.574 (0.302, 1.089) | 0.089 |
| **Smoking** |  |  |  |  |
| No | reference |  | reference |  |
| Yes | 0.389 (0.125, 1.212) | 0.103 | 0.396 (0.113, 1.392) | 0.149 |
| **Alcohol drinking** |  |  |  |  |
| No | reference |  | reference |  |
| Yes | 0.688 (0.361, 1.308) | 0.254 | 0.807 (0.393, 1.657) | 0.560 |
| **Having breakfast 7 days in a week** |  |  |  |  |
| No | reference |  | reference |  |
| Yes | 0.664 (0.444, 0.994) | **0.047** | 0.703 (0.456, 1.083) | 0.110 |
| **Sufficient vegetable intake** |  |  |  |  |
| No | reference |  | reference |  |
| Yes | 0.466 (0.274, 0.794) | **0.005** | 0.519 (0.292, 0.921) | **0.025** |
| **Sufficient fruit intake** |  |  |  |  |
| No | reference |  | reference |  |
| Yes | 0.670 (0.407, 1.101) | 0.114 | 0.957 (0.553, 1.656) | 0.875 |
| **Sufficient sleep** |  |  |  |  |
| No | reference |  | reference |  |
| Yes | 0.794 (0.535, 1.179) | 0.253 | 0.823 (0.535, 1.268) | 0.377 |
| **Excessive screen time on video** |  |  |  |  |
| No | reference |  | reference |  |
| Yes | 0.893 (0.598, 1.334) | 0.580 | 0.890 (0.566, 1.401) | 0.615 |
| **Excessive screen time on electronic game** |  |  |  |  |
| No | reference |  | reference |  |
| Yes | 1.452 (0.978, 2.156) | 0.065 | 1.703 (1.077, 2.694) | **0.023** |
| **Excessive screen time on social media** |  |  |  |  |
| No | reference |  | reference |  |
| Yes | 0.564 (0.379, 0.840) | **0.005** | 0.426 (0.270, 0.673) | **<0.001** |
| **Self-reported obesity** |  |  |  |  |
| No | reference |  | reference |  |
| Yes | 1.150 (0.747, 1.77) | 0.525 | 1.033 (0.657, 1.626) | 0.888 |
| **MTS-A** |  |  |  |  |
| Low | reference |  | reference |  |
| High | 0.476 (0.313, 0.723) | **<0.001** | 0.532 (0.337, 0.838) | **0.006** |
| **Health literacy level** |  |  |  |  |
| Low | reference |  | reference |  |
| High | 0.709 (0.478, 1.053) | 0.088 | 0.948 (0.612, 1.468) | 0.810 |

Sufficient physical activity: Moderate/Vigorous exercise >=60 minutes every day; Physical inactivity: Moderate/Vigorous exercise <60 minutes every day; SES: Socioeconomic Status; Sufficient vegetable intake: Had ≥ 1.5 bowls of vegetables every day; Sufficient fruit intake: Had ≥ 2 servings of fruit every day; Sufficient sleep: Sleep at least 8 hours; MTS-A: Mental Toughness Scale for Adolescents; Health literacy level: Scores for evaluating personal competencies for the access to, understanding of, appraisal of and application of health information in order to make sound decisions in everyday life. Significant variables (p<0.05) were in bold.

Hosmer and Lemeshow goodness of fit (GOF) test:

X-squared = 11.343, df = 8, p-value = 0.183.

CI: confidence interval; OR, odds ratio; aOR, adjusted odds ratio. ORs / aORs > 1 indicate that the target group has higher odds of the outcome (physical inactivity) compared with the reference group,

Supplementary Table 6. Factors associated with physical inactivity frequency in female group

|  | OR (95% CI)* | *P-value* | aOR (95% CI) | *P-value* |
| --- | --- | --- | --- | --- |
| **Age group** |  |  |  |  |
| <13 | reference |  | reference |  |
| 13-14 | 1.683 (0.853, 3.322) | 0.133 | 1.524 (0.743, 3.127) | 0.251 |
| >14 | 1.920 (0.994, 3.709) | 0.052 | 2.491 (1.200, 5.170) | **0.014** |
| **SES** |  |  |  |  |
| Low | reference |  | reference |  |
| Medium | 0.762 (0.325, 1.782) | 0.530 | 1.067 (0.442, 2.578) | 0.885 |
| High | 0.755 (0.315, 1.811) | 0.529 | 1.177 (0.470, 2.949) | 0.728 |
| **Smoking** |  |  |  |  |
| No | reference |  | reference |  |
| Yes | 0.316 (0.066, 1.523) | 0.151 | 0.248 (0.039, 1.577) | 0.140 |
| **Alcohol drinking** |  |  |  |  |
| No | reference |  | reference |  |
| Yes | 0.400 (0.192, 0.833) | **0.014** | 0.251 (0.106, 0.595) | **0.002** |
| **Having breakfast 7 days in a week** |  |  |  |  |
| No | reference |  | reference |  |
| Yes | 1.001 (0.590, 1.698) | 0.998 | 1.147 (0.641, 2.053) | 0.643 |
| **Sufficient vegetable intake** |  |  |  |  |
| No | reference |  | reference |  |
| Yes | 0.572 (0.304, 1.075) | 0.083 | 0.710 (0.360, 1.399) | 0.322 |
| **Sufficient fruit intake** |  |  |  |  |
| No | reference |  | reference |  |
| Yes | 0.545 (0.294, 1.010) | 0.054 | 0.606 (0.307, 1.195) | 0.148 |
| **Sufficient sleep** |  |  |  |  |
| No | reference |  | reference |  |
| Yes | 0.830 (0.488, 1.412) | 0.491 | 0.834 (0.467, 1.490) | 0.539 |
| **Excessive screen time on video** |  |  |  |  |
| No | reference |  | reference |  |
| Yes | 1.233 (0.727, 2.090) | 0.437 | 1.033 (0.561, 1.902) | 0.916 |
| **Excessive screen time on electronic game** |  |  |  |  |
| No | reference |  | reference |  |
| Yes | 2.152 (1.216, 3.808) | **0.008** | 2.448 (1.284, 4.665) | **0.007** |
| **Excessive screen time on social media** |  |  |  |  |
| No | reference |  | reference |  |
| Yes | 0.916 (0.538, 1.562) | 0.749 | 0.634 (0.337, 1.196) | 0.159 |
| **Self-reported obesity** |  |  |  |  |
| No | reference |  | reference |  |
| Yes | 1.564 (0.866, 2.825) | 0.138 | 1.533 (0.817, 2.875) | 0.183 |
| **MTS-A** |  |  |  |  |
| Low | reference |  | reference |  |
| High | 0.665 (0.392, 1.129) | 0.131 | 0.864 (0.477, 1.568) | 0.632 |
| **Health literacy level** |  |  |  |  |
| Low | reference |  | reference |  |
| High | 0.519 (0.299, 0.898) | **0.019** | 0.545 (0.296, 1.003) | 0.051 |

Sufficient physical activity: Moderate/Vigorous exercise >=60 minutes every day; Physical inactivity: Moderate/Vigorous exercise <60 minutes every day; SES: Socioeconomic Status; Sufficient vegetable intake: Had ≥ 1.5 bowls of vegetables every day; Sufficient fruit intake: Had ≥ 2 servings of fruit every day; Sufficient sleep: Sleep at least 8 hours; MTS-A: Mental Toughness Scale for Adolescents; Health literacy level: Scores for evaluating personal competencies for the access to, understanding of, appraisal of and application of health information in order to make sound decisions in everyday life. Significant variables (p<0.05) were in bold.

Hosmer and Lemeshow goodness of fit (GOF) test:

X-squared = 6.6633, df = 8, p-value = 0.5734.

CI: confidence interval; OR, odds ratio; aOR, adjusted odds ratio. ORs / aORs > 1 indicate that the target group has higher odds of the outcome (physical inactivity) compared with the reference group,

Supplementary Figure 1.Q-Q plot for continuous variables
