# Supplementary material for: Exploring Patterns and Associated Factors of Physical Inactivity in Adolescents: A Cross‑Sectional Study
Source: Health Sci Rep. 2026 Aug 19;9(8):e72926. doi: 10.1002/hsr2.72926 (PMC13490853; doi:10.1002/hsr2.72926)
Supplement: Supplementary file 2 [file HSR2-9-e72926-s002.docx]

**Appendix 1 Rationale for covariate selection**

Conceptually important covariates were selected a priori based on prior literature, including socio-demographic factors (e.g., age and sex), dietary behaviours (e.g., fruit and vegetable intake), psychosocial factors, and health literacy, which have been consistently linked to adolescent physical activity.(1-9) Specifically, socio‑demographic factors such as age and sex have been shown to be associated with a higher likelihood of physical inactivity;(1, 2) dietary behaviours, particularly fruit and vegetable intake, have been linked to physical inactivity and tend to cluster with sedentary lifestyle patterns;(3, 4) and psychosocial factors, including mental toughness–related constructs, are associated with lower levels of physical inactivity.(5, 6) In addition, health literacy has been associated with adolescents’ health behaviours, including reduced likelihood of physical inactivity.(7-9)

**Appendix 2 Sensitivity analysis results**

Sensitivity analyses generally supported the robustness of the main findings. First, analyses using continuous measures of MTS‑A and health literacy yielded results broadly consistent with those of the dichotomised models, suggesting that the main associations were not materially influenced by variable categorisation (Supplementary Table 3). Second, sensitivity analyses using multilevel logistic regression with school included as a random intercept produced results consistent with the primary model; the intraclass correlation coefficient (ICC) was 0.101 in the null model and decreased to approximately 0.05 after covariate adjustment, which is within the range reported for adolescent health outcomes in secondary schools (Supplementary Table 4).(10) Finally, multivariable logistic regression models stratified by sex showed patterns largely consistent with the primary analysis, although some differences in effect sizes and statistical significance were observed between males and females. In sex-stratified multivariable models, excessive gaming time was consistently associated with higher odds of physical inactivity in both males (aOR=1.703, 95% CI: 1.077–2.694, P=0.023) and females (aOR=2.448, 95% CI: 1.284–4.665, P=0.007), whereas higher mental toughness (aOR=0.532, 95% CI: 0.337–0.838, P=0.006), sufficient vegetable intake (aOR=0.519, 95% CI: 0.292–0.921, P=0.025), and excessive social media screen time (aOR=0.426, 95% CI: 0.270–0.673, P<0.001) were significant only among males; age >14 was significant only among females (aOR=2.491, 95% CI:1.200–5.170, P=0.014), and alcohol use showed an inverse association primarily in females (aOR=0.251, 95% CI: 0.106–0.595, P=0.002) (Supplementary Tables 5–6).

**References**

1.	Guthold R, Stevens GA, Riley LM, Bull FC. Global trends in insufficient physical activity among adolescents: a pooled analysis of 298 population-based surveys with 1· 6 million participants. The Lancet Child & Adolescent Health. 2020;4(1):23-35.

2.	Mielke GI, da Silva ICM, Kolbe-Alexander TL, Brown WJ. Shifting the Physical Inactivity Curve Worldwide by Closing the Gender Gap. Sports Medicine. 2018;48(2):481-9.

3.	Silva DA, Silva RJ. Association between physical activity level and consumption of fruit and vegetables among adolescents in northeast Brazil. Rev Paul Pediatr. 2015;33(2):167-73.

4.	Darfour-Oduro SA, Andrade JE, Grigsby-Toussaint DS. Do Fruit and Vegetable Policies, Socio-Environmental Factors, and Physical Activity Influence Fruit and Vegetable Intake Among Adolescents? Journal of Adolescent Health. 2020;66(2):172-80.

5.	Hale GE, Colquhoun L, Lancastle D, Lewis N, Tyson PJ. Review: Physical activity interventions for the mental health and well-being of adolescents – a systematic review. Child and Adolescent Mental Health. 2021;26(4):357-68.

6.	Wassenaar TM, Wheatley CM, Beale N, Nichols T, Salvan P, Meaney A, et al. The effect of a one-year vigorous physical activity intervention on fitness, cognitive performance and mental health in young adolescents: the Fit to Study cluster randomised controlled trial. International Journal of Behavioral Nutrition and Physical Activity. 2021;18(1):47.

7.	Fleary SA, Joseph P, Pappagianopoulos JE. Adolescent health literacy and health behaviors: A systematic review. Journal of adolescence. 2018;62:116-27.

8.	Huang J, Cheung CK, Chan SC, Pang WS, Chow SH, Li QH, et al. Factors associated with physical inactivity among the pre-school children: A cohort of 1681 participants. Journal of Paediatrics and Child Health. 2023;59(10):1152-9.

9.	Martins LCG, Lopes MVO, Diniz CM, Guedes NG. The factors related to a sedentary lifestyle: A meta-analysis review. Journal of Advanced Nursing. 2021;77(3):1188-205.

10.	Shackleton N, Hale D, Bonell C, Viner RM. Intraclass correlation values for adolescent health outcomes in secondary schools in 21 European countries. SSM Population Health. 2016;2:217-25.
